# A Underwater Sequence Image Dataset for Sharpness and Color Analysis

**DOI:** 10.3390/s22093550

**Published:** 2022-05-07

**Authors:** Miao Yang, Ge Yin, Haiwen Wang, Jinnai Dong, Zhuoran Xie, Bing Zheng

**Affiliations:** 1 School of Electronic Engineering, Jiangsu Ocean University, Lianyungang 222005, China; lemonmiao@gmail.com (M.Y.); w615933060@163.com (H.W.); jinnaidong@outlook.com (J.D.); jouxiezhuoran@outlook.com (Z.X.); 2 Department of Information Science and Engineering, Ocean University of China, Qingdao 266001, China; bingzh@ouc.edu.cn

**Keywords:** underwater dataset, underwater image quality, sequence image

## Abstract

The complex underwater environment usually leads to the problem of quality degradation in underwater images, and the distortion of sharpness and color are the main factors to the quality of underwater images. The paper discloses an underwater sequence image dataset called TankImage-I with gradually changing sharpness and color distortion collected in a pool. TankImage-I contains two plane targets, a total of 78 images. It includes two lighting conditions and three different water transparency. The imaging distance is also changed during the photographing process. The paper introduces the relevant details of the photographing process, and provides the measurement results of the sharpness and color distortion of the sequence images. In addition, we verify the performance of 14 image quality assessment methods on TankImage-I, and analyze the results of 14 image quality assessment methods from the aspects of sharpness and color, which provides a reference for the design and improvement of underwater image quality assessment algorithm and underwater imaging system design.

## 1. Introduction

Underwater vision is an important basis for scientific research in ocean exploration, marine biological surveys, and underwater engineering monitoring [1,2,3]. Due to the absorption and scattering effects of water bodies and substances in water and the complexity of the underwater environment, there is usually quality degradation in optical underwater images [4,5,6,7]. Therefore, underwater image/video quality evaluation is of great significance for high-quality image screening and comparison of underwater image enhancement/restoration results. The analysis of image quality and the design of corresponding algorithm are closely related to the dataset with known real quality. The natural image dataset with sequence distortion can be used to verify the consistency of image quality assessment (IQA) method and distortion level, and can provide reference for the improvement and design of IQA.

Different distortion level images cannot be obtained in a real underwater environment, so acquiring image sequences by controlled pool imaging conditions is a common method, such as the Turbid dataset [8], the OUC-Vision dataset [9], and the NWPU dataset [10], but these datasets do not consider the effect of imaging distance variation on underwater image quality, and did not provide measurements of image sharpness and color distortion, making it difficult to fully validate the performance of the image quality evaluation method in terms of sharpness and color distortion of sequential images.

This paper provides an underwater sequence image dataset called TankImage-I with gradually sharpness and color distortion, and provides the corresponding sharpness and color distortion measurements. Two plane targets are included in TankImage-I: the ColorChecker card and the SFR board, with a total of 78 images. We photograph in three different transparent water bodies. The light source conditions include underwater natural light and artificial light source. During the photographing process, the imaging distance is changed by moving the target position. As far as we know, TankImage-I is the underwater image sequence with the most comprehensive experimental conditions in underwater image quality measurement. This is conducive to underwater image enhancement and image quality evaluation methods to measure sharpness and color respectively. In addition, we conducted several experiments of image quality evaluation methods on this image sequence and analyzed the results of the image quality evaluation methods from the two aspects of sharpness and color, which provide guidance for the design and improvement of subsequent underwater image quality evaluation algorithms and underwater image enhancement algorithms in terms of sharpness and color.

## 2. Related Work

### 2.1. Underwater Image Database

At present, there are many real underwater image datasets, such as fish4knowledge dataset for underwater target detection and recognition [11], which contains a variety of fish images; Islam et al. built an underwater image database EUVP [12] for image enhancement. The EUVP includes 10,000 pairs of images and 25,000 unpaired images. Unpaired images are taken by seven different cameras. The photographing environment includes the marine environment under different visibility conditions. The paired images are generated by CycleGAN [13], forming an image pair with unpaired images; Li et al. similarly built a database for underwater image enhancement (underwater image enhancement benchmark, UIEB) [14] which included 950 real world underwater images, of which 890 were enhanced by the authors, and the remaining 60 underwater images that could not obtain satisfactory enhanced images were regarded as challenging data.

However, most of the underwater images in these underwater real image datasets only include one scene, so it is difficult to measure the changes of sharpness and color distortion in the image alone. Therefore, there are also some underwater image datasets with different distortions obtained by controlling the experimental conditions, such as OUC-VISION [9], a large underwater database for underwater salient target detection or saliency detection proposed by Jian et al. which contains 4400 images of 220 objects, each using four pose variations (front, opposite, left and right side views) and five spatial positions (top left, top right, center, bottom left and bottom right) were photographed, while OUC-VISION changed the transparency of the water body by adding soil to the water. In terms of light source, the combination of three LEDs is used to simulate four lighting conditions, However, the imaging distance in OUC-VISION is fixed, and the influence of the change of imaging distance on underwater image quality cannot be simulated; Duarte et al. proposed the TURBID sequence images [8], where the photographs of real underwater images were taken and placed in a water tank, and then the turbidity of the water was changed by controlling the milk added to the tank, Duarte et al. proposed the TURBID-3D dataset based on the TURBID sequence image set, and the experimental objects in TURBID-3D were increased with 3D objects such as rocks on the sea floor, corals. The TURBID and TURBID-3D datasets only changed the transparency of the water body, and the imaging distance and illumination conditions did not change. The experimental conditions of the above underwater sequence image sets are not comprehensive enough, and there is a lack of measurement results of sharpness and color distortion, so there are limitations in verifying the performance of underwater quality evaluation algorithms and image enhancement algorithms in sharpness and color distortion. Camilo et al. [15] proposed an underwater sequence image set UIDLEIA-DATABASE with real quality score. The targets include five colors, and the photographing environment includes different changes of water turbidity and imaging distance. The image quality score is obtained by single stimulation method. However, the illumination conditions of underwater environment are not changed in UIDLEIA-DATABASE, and the change of sharpness distortion is not measured. Table 1 gives a brief summary of the above databases.

### 2.2. Image Quality Assessment Method

Sheikh et al. first applied natural scenes statistics (NSS) in the field of image quality evaluation and proposed the NSS-based method JP2KNR for blind IQA (BIQA) [16], JP2KNR showed that human perception of image quality and perception of distortion is related to the natural statistics of images, then more and more methods based on NSS features have been developed subsequently. Moorthy et al. proposed DIIVINE [17] based on identifying the type of image distortion, DIIVINE uses wavelet decomposition at two scales to obtain a directional bandpass response and then extracts a series of statistical features using the obtained subband coefficients. Mittal et al. proposed BRISQUE [18] to obtain the fitted coefficients by extracting the product of the mean subtracted contrast normalized (MSCN) coefficients of the image and the adjacent coefficients of the MSCN, and then fitting the above coefficients using the generalized gaussian distribution (GGD) [19] as quality related features. Saad et al. proposed a method BLIINDS2 based on DCT domain [20]. BLIINDS2 learns the mapping from quality features to quality scores through probability prediction model; Xue et al. proposed BIQA model GM-LOG based on gradient magnitude (GM) and response of Laplacian of Gaussian (LOG) [21]; Zhang et al. proposed the evaluation method ILNIQE integrating multiple NSS features [22]. Inspired by ILNIQE, Liu et al. proposed SNPNIQE [23] to measure the degradation of image quality in terms of changes in structure, naturalness, and perceptual quality, where changes in structure are represented by deviations in phase congruency (PC) and gradient distribution, and changes in naturalness are characterized by the product of MSCN and the products of pairs of the adjacent MSCN coefficients, using a sparse model to simulate the changes in perception by human vision. Liu et al. [24] proposed a NSS and Perceptual characteristics-based Quality Index (NPQI), which extracts a set of quality-aware NSS and perceptual characteristics-related features, and then build a pristine multivariate Gaussian (MVG) model to infer the image quality.

In addition to the above methods based on NSS features, Ye et al. proposed a codebook based method CORNIA [25], which extracts the mean value of image blocks for normalization, and performs ZCA (zero components analysis) whitening, then uses the standardized image blocks as local features for the construction of codebook; In HOSA [26] proposed by Xu et al., image blocks are also extracted to establish codebook. Compared with CORNIA, HOSA not only calculates the mean value of clusters, but also calculates the two high-order features of variance and skewness for clustering; Liu et al. proposed SSEQ [27] to extract the phase consistency and spatial entropy of the image as image features; Yang et al. proposed MsKLT [28] based on the KLT transform, and MsKLT extracts the KLT coefficients of the image and uses GGD to obtain the fitting parameters as quality related features.

The IQA methods described above were all experimented on natural image datasets, so the performance of these methods in underwater images with complex distortion needs to be confirmed.

### 2.3. Underwater Image Quality Assessment Method

Underwater IQA methods mostly focus on evaluating the enhancement and recovery of grayscale underwater images [29,30,31,32]. For example, Schechner and Karpel et al. [33] analyzed the physical impact of underwater visibility decline and restored the image by enhancing the contrast of underwater image; Hou [34] et al. and others proposed an image sharpness evaluation standard based on weighted gray scale angle (GSA) for underwater target images with noise. Arredondo and lebart et al. [35] proposed a method for quantitative evaluation of underwater noise in underwater video images.

For underwater color images, Karen et al. [36] proposed an underwater IQA method UIQM (underwater image quality measure), which combines color measurement, sharpness measurement and contrast measurement as a basis for evaluating the quality of underwater images. Yang et al. proposed a UCIQE (underwater color image quality evaluation) [37] metric for underwater color image quality evaluation, using CIELab spatial chroma, saturation and contrast as quality measures, and uses the obtained MOS to fit the weighting coefficient by multiple linear regression; The FDUM [38] proposed by Yang et al. also extracts the component values of three aspects of underwater image: chroma, contrast and sharpness. For the low contrast distortion caused by backscattering, FDUM proposes a dark channel prior weighted contrast measure to enhance the discrimination ability of the original contrast measurement, and the same sharpness, color and contrast components are extracted and then weighted.

From the above underwater IQA methods, it can be seen that sharpness and color are closely related to underwater image quality, so an underwater image quality dataset with gradual changes in sharpness and color distortion is important for underwater image quality evaluation methods.

## 3. TankImage-I Environment Setup and Analysis

### 3.1. Camera System and Lighting System

**Figure 1:**
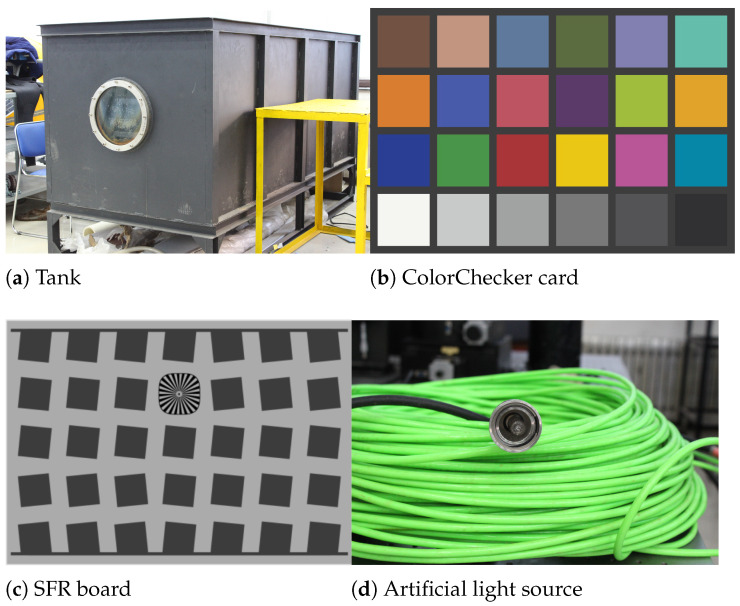
Experimental environment and targets.

The tank for photographing was 2.53 m long, 1.02 m wide and 1.03 m high, with observation windows on both sides. The photographing targets of the water tank environment are SFR board and ColorChecker card (21.59 × 27.94 cm), the tank and targets are shown in Figure 1a–c. The camera selects OTI-UWC-325/P/E color camera. We choose three kinds of water quality with different transparency for shooting: clear, medium turbid and turbid. The transparency of water body measured by blackboard method [39] is 325 cm (clear), 182 cm (medium turbid) and 85 cm (turbid). We use a 150 W halogen lamp as the artificial light source. As shown in Figure 1d, the artificial light source is placed 50 cm away from the camera.

### 3.2. Imaging Distance Setting

The water depth in the tank was 90 cm, and the target was placed 45 cm from the surface of the water body. Keeping the camera and lights stationary, the target was moved every 10 cm, TankImage-I contains 12 sequences, a total of 78 images. The specific imaging distance and other information are shown in Table 2. The higher the transparency in Table 2, the higher the degree of visibility, i.e., the highest degree of visibility when the transparency is 325 cm, the next highest transparency is 182 cm. The worst visibility is at 85 cm.

### 3.3. Imatest Quality Evaluation Software Evaluation

We use Imatest software to measure the sharpness and color distortion of the image in TankImage-I. Imatest software tests cameras and imaging systems by comparing the differences between standard images from a graphics card and captured test images. Imatest includes modules such as SFR, Colorcheck, and Stepchart. In order to measure the change of sharpness and color in underwater images as the experimental conditions change, two plane targets, the SFR board and the ColorChecker card, are selected as imaging targets in this paper. The SFR plus module automatically analyzes the sharpness, field of view, aberrations and other image quality parameters of the SFR board; the Colorcheck module analyzes the color accuracy, tonal response, gamma, signal-to-noise ratio and other parameters of the ColorChecker card.

The sharpness in the SFR plus module can be obtained by measuring the spatial frequency response (SFR), also called the modulation transfer function (MTF). We chose to use the value of MTF50 as the sharpness indicator since the 50% MTF is in good agreement with the human vision of the sharpness results. Table 3 shows the results of the SFR board images analyzed using the SFR plus module. (where “∖” indicates that no image was taken at that distance).

The color error $\Delta {E^{*}}_{\mathrm{ab}}$ between the standard ColorChecker card and the captured ColorChecker card image on the CIELAB color space is calculated in the Colorcheck module. $\Delta {E^{*}}_{\mathrm{ab}}$ is calculated as shown in Equation (1).

(1) $$\Delta {E^{*}}_{\mathrm{ab}}={\left({\left(L_{2}-L_{1}\right)}^{2}+{\left(a_{2}-a_{1}\right)}^{2}+{\left(b_{2}-b_{1}\right)}^{2}\right)}^{1/2}$$

where $L_{1}$, $L_{2}$ represent the luminance of the standard ColorChecker test card and the experimentally taken ColorChecker test card, respectively, $a_{2}$ and $a_{2}$ are the values of the green red channel in CIELAB space, and $b_{1}$ and $b_{1}$ are the values of the blue yellow channel. and we choose $\Delta {E^{*}}_{\mathrm{ab}}$ as the color distortion index of the image. Table 4 shows the results of the ColorChecker test card images analyzed using the Colorcheck module.

## 4. Experiment and Analysis

### 4.1. Result Analysis of Image Quality Evaluation Method on Tankimage-I

**Figure 2:**
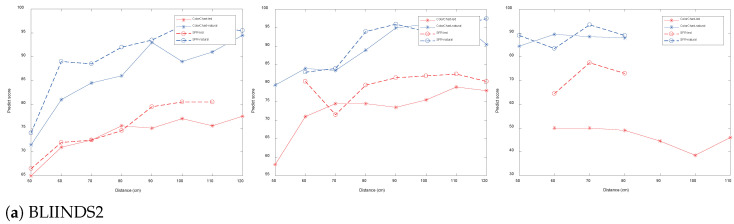

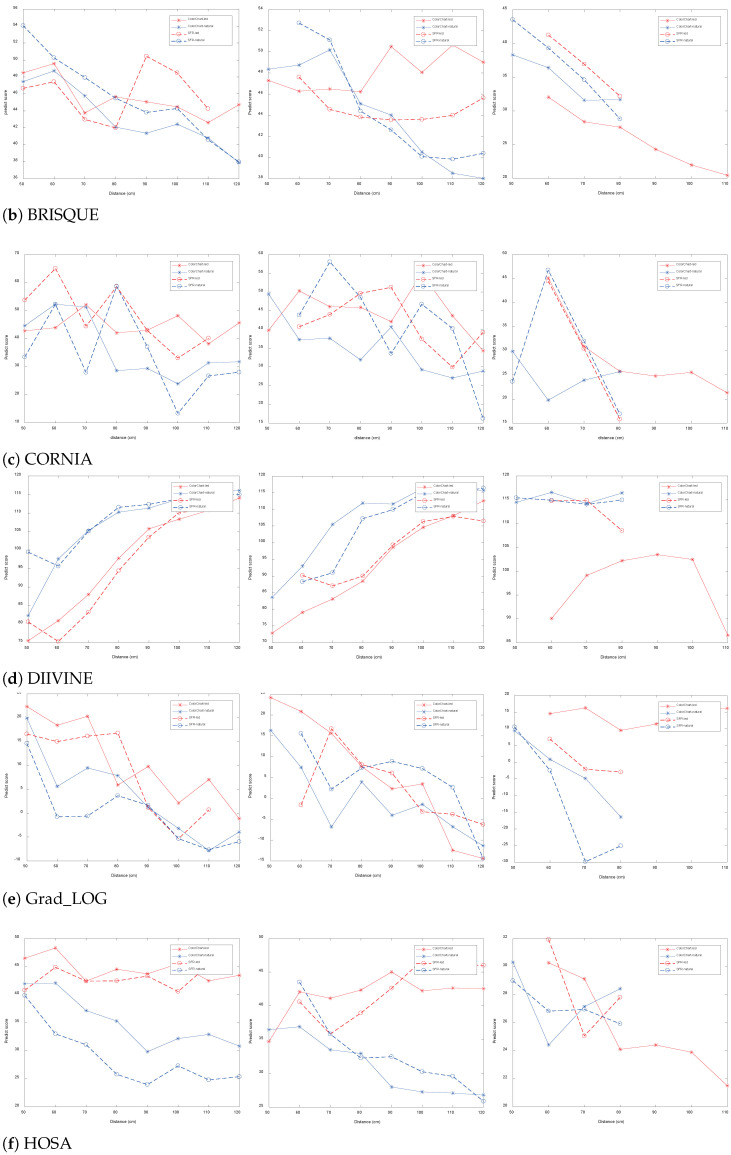

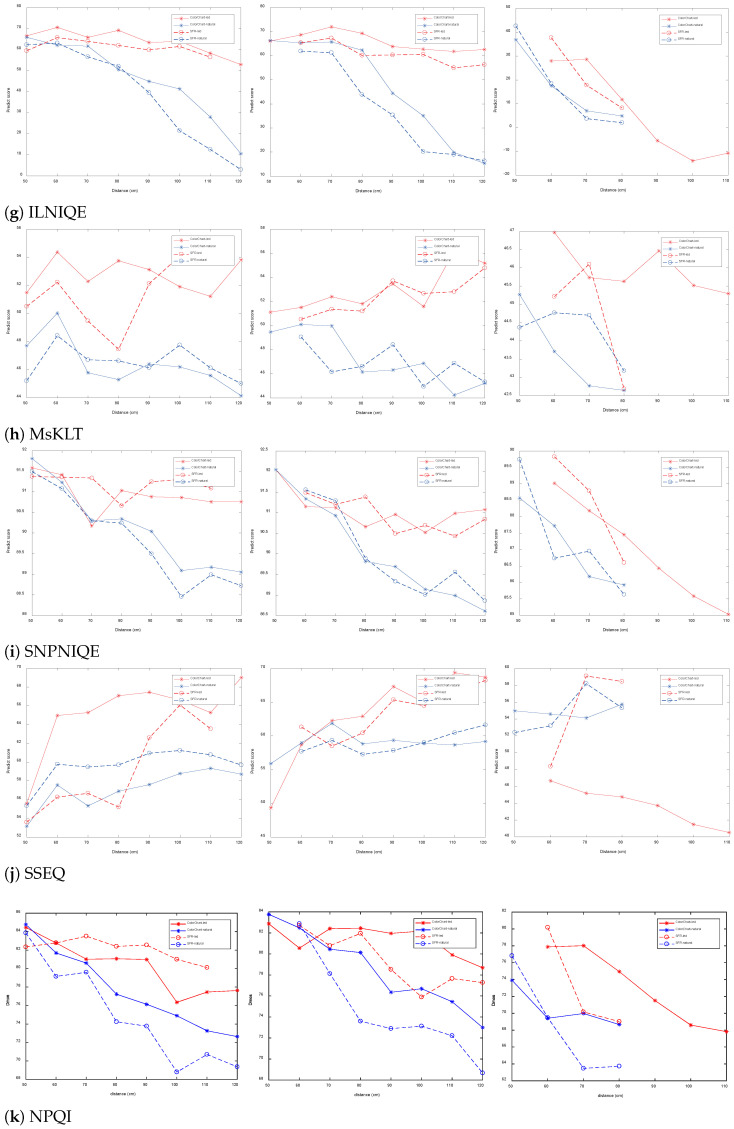

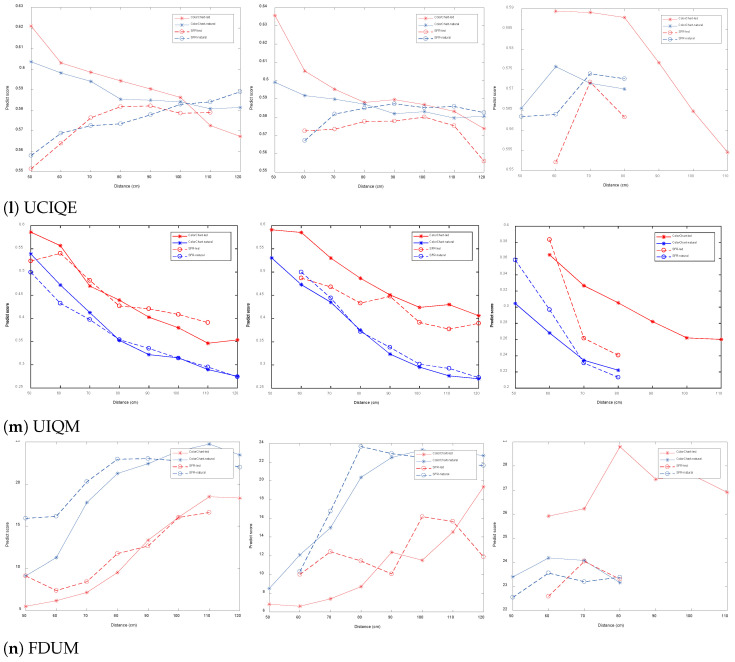
Predict score curves of each quality evaluation method on Tankimage-I.

In this paper, we select 11 natural image quality assessment methods: BLIINDS2 [20], BRISQUE [18], CORNIA [25], DIIVINE [17], Grad_Log [21], ILNIQE [22], HOSA [26], SNPNIQE [23], SSEQ [27], MSKLT [28] and NPQI [24]; three underwater image quality assessment methods: UCIQE [37], UIQM [36] and FDUM [38], a total of 14 methods to evaluate the quality of each sequence image set in TankImage-I. The prediction quality score curves of each method are shown in Figure 2, and the abscissa is imaging distance. The water transparency of the image in the leftmost column in Figure 2 is 325 cm, the water transparency of the image in the middle column is 182 cm, and the water transparency of the image in the rightmost column is 85 cm.

**Figure 3:**
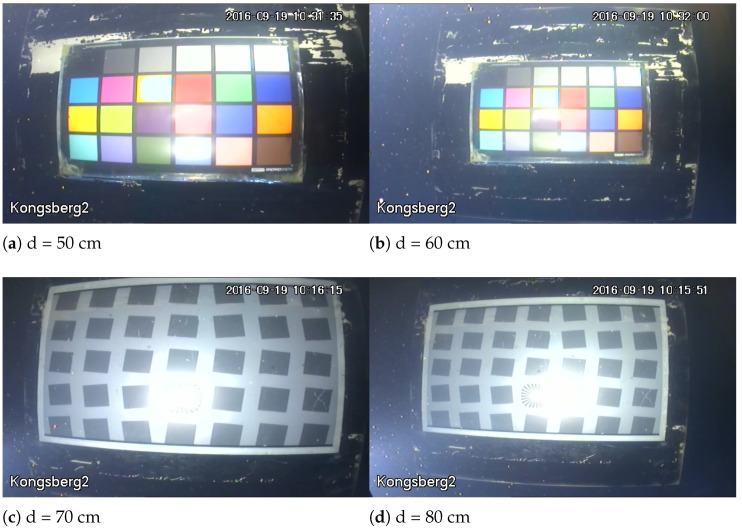
The transparency of water body is 325 cm, and some images under artificial light source.

**Figure 4:**
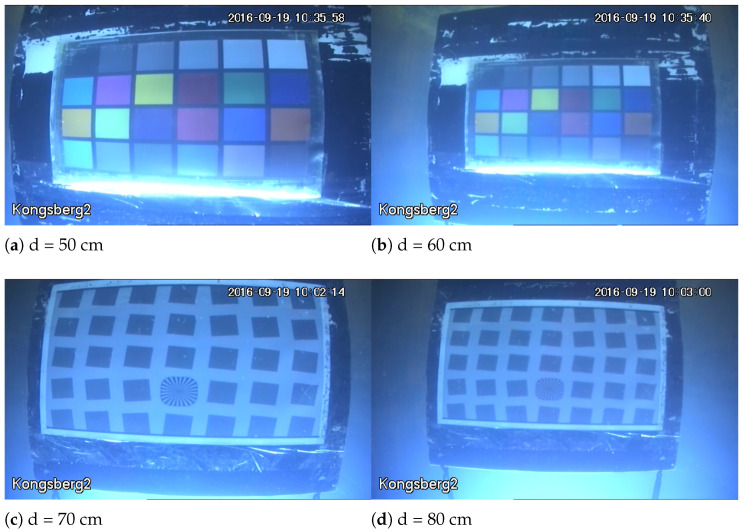
The transparency of the water body is 325 cm, which is part of the images under natural light.

For the SFR board image, under the water transparency of 325 cm and artificial light source, as shown in Figure 3, when the imaging distance is small there is a bright reflection area in the image, which makes the local clarity of the image change, and with the increase of the imaging distance, the presence of suspended matter in the water makes the noise in the image, and the backward scattering becomes the main attenuation component, the evaluation result of UCIQE is in good agreement with the change of blurring degree of the SFR board. When the lighting condition is natural light, as shown in Figure 4, foggy blur and low contrast distortion caused by scattering are mainly present in the image. At this time, the scores of BRISQUE, HOSA and UCIQE are in good agreement with the change of blurring degree of SFR board. And the distortion when the water transparency is 182 cm is similar to the distortion when the water transparency is 325 cm. When the imaging distance is larger, the noise in the image under artificial light source is more serious, and the evaluation results of BRISQUE, HOSA and UIQM have better consistency in the change of blurring degree of SFR plate. And the consistency of the blurring degree change of SFR plate is better for the evaluation results of BRISQUE, SNPNIQE and UCIQE under natural light. (d is the imaging distance)

For ColorChecker card images, the evaluation results of MsKLT and ILNIQE were in good agreement with the change of color distortion degree under the water transparency of 325 cm and artificial light source; the evaluation results of UCIQE were in good agreement with the change of color distortion degree under natural light. While the transparency of water body is 82 cm, the evaluation results of ILNIQE, UCIQE and UIQM under artificial light source are in better agreement with the change of color distortion degree.

### 4.2. Consistency Analysis of Image Quality Evaluation Methods and Sharpness and Color Distortion

**Figure 5:**
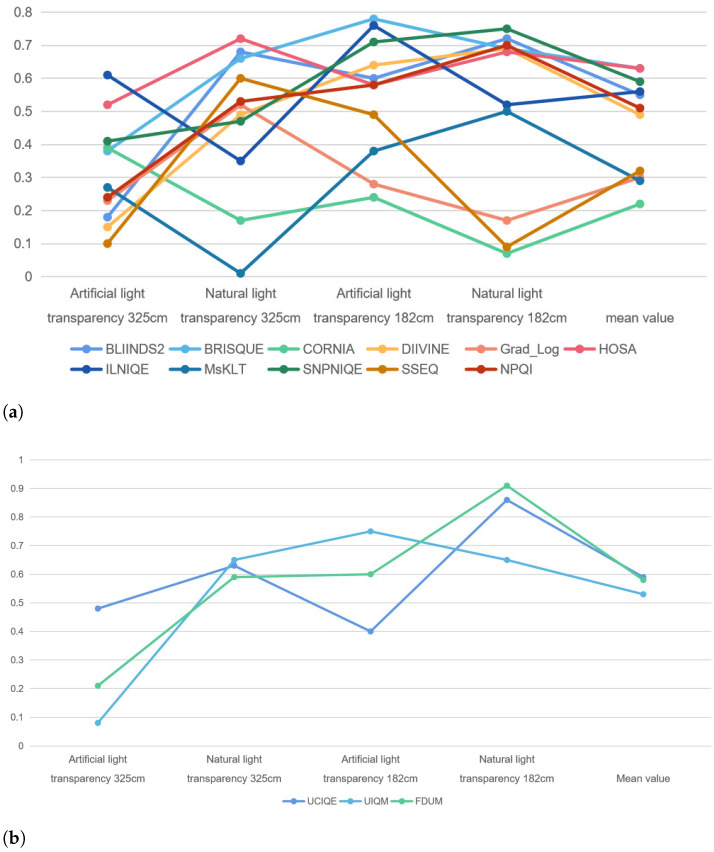
PLCC curve between IQA predict results and MTF50 value. (**a**) PLCC curve between results of natural IQA methods and MTF50 value, (**b**) PLCC curve between results of underwater IQA methods and MTF50 value.

**Figure 6:**
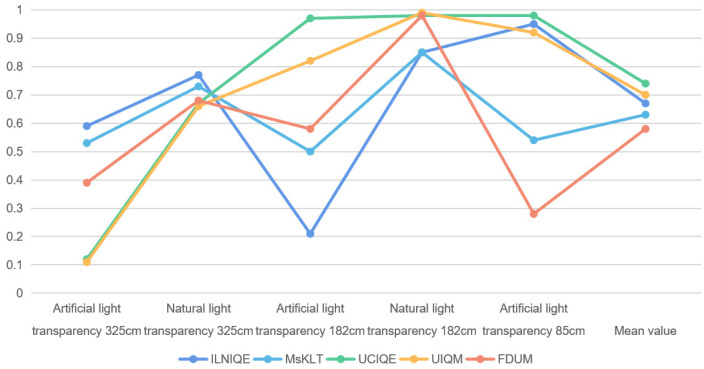
PLCC curve between IQA predict results and $\Delta {E^{*}}_{\mathrm{ab}}$ value.

In order to further analyze the relationship between the evaluation results of the 14 quality evaluation methods on TankImage-I and the sharpness and color distortion, we calculated the PLCC values between the evaluation results of the above quality evaluation methods on the SFR board and the MTF50 measured under the corresponding experimental conditions, and the results are shown in Table 5. (SFR images at 85 cm transparency are too few, so they are not calculated separately). In addition, we also calculated the PLCC values between the evaluation results of the five color image quality evaluation methods on the ColorCherker card and the $\Delta {E^{*}}_{\mathrm{ab}}$ measured under the corresponding experimental conditions, and the results are shown in Table 6. The corresponding curves of PLCC between IQA prediction results and MTF50, and PLCC between IQA prediction results and $\Delta {E^{*}}_{\mathrm{ab}}$ are shown in Figure 5 and Figure 6.

**Figure 7:**
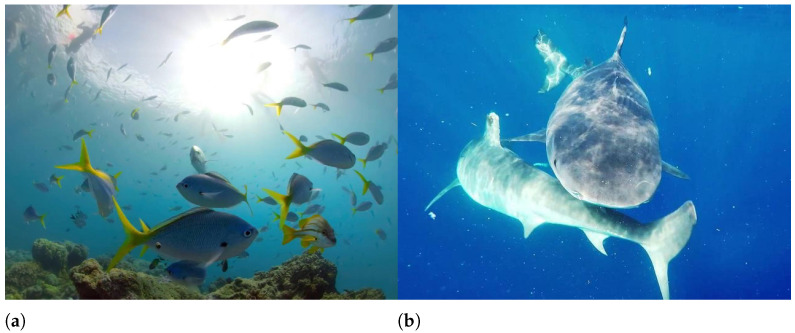
Bliinds2, UIQM and FDUM misjudgment example. (**a**) Bliinds2(DMOS)=20 UIQM = 0.93 FDUM = 0.43, (**b**) Bliinds2(DMOS) = 29 UIQM = 0.91 FDUM = 0.06.

From Figure 5, it can be seen that the average PLCC values of BLIINDS2, BRISQUE, HOSA, ILNIQE, NPQI and SNPNIQE in the natural image quality evaluation methods are all over 0.5, where BLIINDS2 has a PLCC value less than 0.2 under the transparency of 325 cm and artificial light source from Table 5. When the imaging distance is small, as shown in Figure 3, it can be seen that there is a bright reflection on the SFR board, and the sharpness around the high bright reflection area changes, which affects the score of BLIINDS2. As shown in Figure 7, there is a highlighted area in (a), and BLIINDS2 prediction score (The higher the DMOS value, the worse the image quality) misjudged the relative quality of image pairs of (a) and (b). Therefore, the transparency of BLIINDS2, UIQM and FDUM in Figure 2 is 325 cm, and the score curve under artificial light source is inconsistent with the change of sharpness distortion.

**Figure 8:**
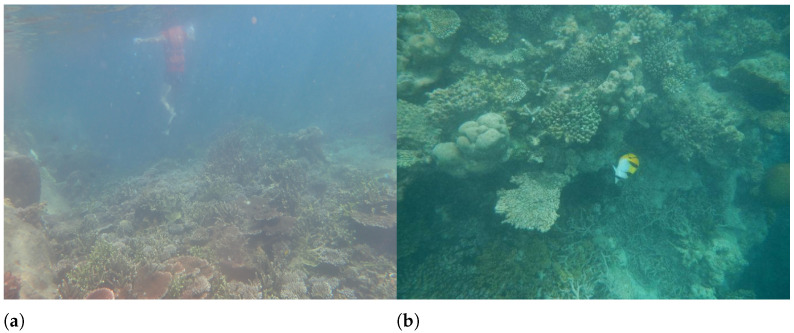
Bliinds2 and FDUM misjudgment example. (**a**) Bliinds2(DMOS) = 8 FDUM = 0.31, (**b**) Bliinds2(DMOS) = 15 FDUM = 0.24.

**Figure 9:**
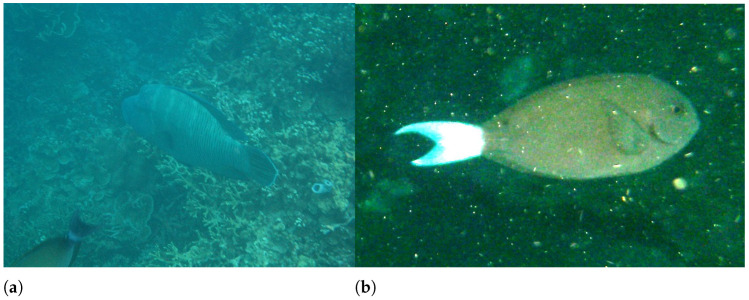
SNPNIQE misjudgment example. (**a**) SNPNIQE(DMOS) = 7.40, (**b**) SNPNIQE(DMOS) = 9.47.

In addition, although the PLCC values between BLIINDS2 score and MTF50 in natural light exceed 0.6 for both transparency of 325 cm and 182 cm, BLIINDS2 score curve at transparency of 325 cm and natural light are not consistent with the change in the degree of sharpness distortion. This may be due to the fact that the DCT coefficients extracted by BLIINDS2 do not adequately represent the distortion variation in underwater images, which are mainly affected by backscatter. As shown in Figure 8, both (a) and (b) have blurring and low contrast caused by backward scattering, and the distortion is more severe in (b) than in (a), while BLIINDS2 prediction score misjudged the relative quality of image pairs of (a) and (b), and BLIINDS2 also averages the extracted local features, which can cause further damage to the performance of BLIINDS2. Therefore, when the transparency is 325cm and the illumination condition is natural light, the predict result of BLIINDS2 is inconsistent with the change of sharpness distortion. The phase consistency feature extracted from SNPNIQE is easily affected by noise, which may cause the score of SNPNIQE under artificial light source to be inconsistent with the change of sharpness distortion, as shown in Figure 9.

From Figure 6, for the natural IQA methods, when the water transparency is 325 cm and under the artificial light source, PLCC value between the prediction score of ILNIQE and $\Delta {E^{*}}_{\mathrm{ab}}$ are large, which means ILNIQE has a good assessment performance on the color evaluation of underwater image at this time. PLCC value between the predicted scores of ILNIQE and MsKLT under natural light and the corresponding $\Delta {E^{*}}_{\mathrm{ab}}$ exceed 0.7, that is, the predicted results of ILNIQE and MsKLT are in good agreement with the change of color distortion degree of underwater image. When the water transparency is 182 cm, it can be seen from Table 6 that the PLCC between the predicted score of ILNIQE and the measured value of color distortion under artificial light source is only 0.21. At this time, the backscattering of suspended solids in the underwater background makes noise exist in the image, and ILNIQE is sensitive to noise, which may affects the prediction results of ILNIQE.

For the underwater IQA methods UCIQE and UIQM, when the water transparency is 325 cm and under the artificial light source, the PLCC value between the prediction scores and $\Delta {E^{*}}_{\mathrm{ab}}$ are smaller, which may be due to the existence of highlighted areas in the image and affects the calculation results of contrast components in UCIQE and UIQM.

Therefore, in relatively clear water and under artificial light source, ILNIQE can be considered to evaluate the sharpness and color of underwater image, while UIQM and UCIQE under natural light are more suitable to evaluate the sharpness and color of underwater image. In the turbid water body, ILNIQE and UIQM are more suitable to evaluate the definition and color of underwater image under artificial light source, while UCIQE and FDUM are more suitable to evaluate the sharpness and color of underwater image under natural light.

### 4.3. Underwater Real Image Database Experiment

**Figure 10:**
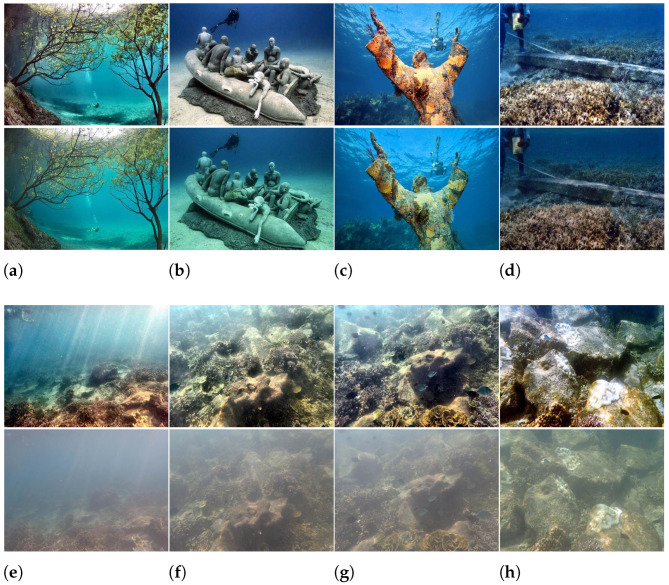
(**a**–**h**) are image pairs selected from UIEB, in which the upper image of each pair is the image after enhancement and the lower image is the image before enhancement.

UIEB [14] database is a database of 950 real-world underwater images. The author enhanced 890 of them and gave the enhanced images. In addition, 60 images with unsatisfactory enhancement effect are regarded as challenging by the author. We selected 8 pair of images, including the original image and the enhanced image, as shown in Figure 10. Among them, the enhanced image in (a)–(d) is better than the image before the enhancement in terms of color, and the enhanced image in (e)–(h) is better than the image before the enhancement in terms of sharpness and contrast. In order to further verify our analysis on the sharpness and color of IQA methods, we analyzed the accuracy results of each IQA method on these 8 image pairs. The results are shown in Table 7. (“T” indicates that the result of the image quality evaluation method correctly judges the relative quality of the image pair, “F” indicates that the result of the image quality evaluation method misjudges the relative quality of the image pair.)

From the Table 7, it can be seen that the accuracy of all three underwater image quality evaluation methods reached 100%, and the accuracy of ILNIQE also reached 100%, because the features extracted by ILNIQE are very rich and include a variety of statistical features. And BLIINDS2 has the lowest accuracy rate. From the results of accuracy, we can see that the pairs with wrong judgment are basically concentrated in (a)–(d), and these pairs have significantly improved the color after enhancement, which indicates that these evaluation methods do not fully utilize the color information of the image, and there is room for further improvement in the processing and utilization of color information in future underwater image quality evaluation methods.

## 5. Conclusions

In this paper, underwater sequence images with gradual changes in sharpness and color distortion were obtained by controlling the experimental conditions and environment, and provides the analysis results of the sharpness and color distortion of the sequence images obtained by using the image quality test software imatest. In addition, the commonly used image quality evaluation methods in 13 are evaluated on each sequence image, and the experimental results are analyzed and further validated, which provides a reference for the design of future algorithms for underwater image quality evaluation, image enhancement, and also provides some ideas for the improvement of the currently existing quality evaluation algorithms.

## Figures and Tables

**Table 1:** Comparison of partial underwater image datasets.

Dataset	Year	Image Number	Target	Imaging Distance	Water Transparency	Illumination Conditions	Distortion Measurement
Fish4Knowlege	2013	27,370	Fishes	Fixed value	Natural transparency of water body	Underwater natural light	None
TURBID	2015	80	Underwater scenes and artifacts	Fixed value	Control the addition of milk to water	Artificial light source	None
TURBID-3D	2016	82	Underwater scenes and artifacts	Fixed value	Control the addition of milk to water	Artificial light source	None
OUC-Vision	2017	4400	Stones and other artifacts	Fixed value	Controls the amount of soil added to the water body	Underwater natural light and artificial light source	None
UIDLEIA-DATABASE	2019	135	Different colors artifacts	Changed	Controls the amount of green tea added to the water body	Fixed	None
EUVP	2020	10,000 image pairs + 25,000 non image pairs	Underwater creatures such as stones and fish	Fixed value	Natural transparency of water body	Underwater natural light	None

**Table 2:** Relevant information of images in Tankimage-I.

Sequence	Number of Images	Target	Turbidty (cm)	With Artificial Light Source or Not	Min Distance (cm)	Max Distance (cm)	Distance Interval (cm)
Sequence1	7	SFR	325	Y	50	110	10
Sequence2	8	SFR	325	N	50	120	10
Sequence3	8	Colorchart	325	Y	50	120	10
Sequence4	8	Colorchart	325	N	50	120	10
Sequence5	7	SFR	182	Y	60	120	10
Sequence6	7	SFR	182	N	60	120	10
Sequence7	8	Colorchart	182	Y	50	120	10
Sequence8	8	Colorchart	182	N	50	120	10
Sequence9	3	SFR	85	Y	60	100	10
Sequence10	4	SFR	85	N	50	100	10
Sequence11	6	Colorchart	85	Y	60	110	10
Sequence12	4	Colorchart	85	N	50	80	10

**Table 3:** Analysis results of SFR plus module on SFR board.

Transparency	Distance from Camera (cm)	Artificial Light Source	MTF50 (LW/PH)	Distance from Camera (cm)	Artificial Light Source	MTF50 (LW/PH)
325 cm	50	Yes	192.3	50	No	148.2
	60		260.5	60		127.5
	70		245.1	70		120.8
	80		256.8	80		116.2
	90		227.1	90		118.6
	100		224.8	100		128.3
	110		260.6	110		133.3
	∖		∖	120		109.1
182 cm	60	Yes	236.9	60	No	146.9
	70		232.4	70		132.9
	80		208.4	80		117.8
	90		210.0	90		120.0
	100		206.3	100		117.3
	110		196.7	110		131.6
	120		222.1	120		127.8
85 cm	60	Yes	74.3	50	No	96.2
	70		79.2	60		97.1
	80		47.0	70		51.8
	∖		∖	80		46.0

**Table 4:** Analysis results of ColorCheck module on ColorCheck card.

Transparency	Distance from Camera (cm)	Artificial Light Source	$\Delta {E^{*}}_{\mathrm{ab}}$	Distance from Camera (cm)	Artificial Light Source	$\Delta {E^{*}}_{\mathrm{ab}}$
325 cm	50	Yes	34.9	50	No	61.0
	60		43.7	60		55.6
	70		44.8	70		58.1
	80		47.7	80		60.1
	90		40.8	90		61.3
	100		38.6	100		62.6
	110		38.2	110		63.5
	120		37.0	120		63.7
182 cm	50	Yes	27.8	50	No	51.3
	60		39.5	60		54.2
	70		45.8	70		56.8
	80		50.0	80		58.6
	90		47.7	90		61.3
	100		48.6	100		62.6
	110		47.9	110		63.5
	120		49.7	120		63.7
85 cm	60	Yes	60.2	50	No	50.2
	70		60.4	60		50.9
	80		57.0	70		52.7
	90		51.4	80		53.6
	100		47.7	∖		∖
	110		43.9	∖		∖

**Table 5:** PLCC between each method and MTF50 under different experimental conditions.

Water Transparency	325 cm	325 cm	182 cm	182 cm	Mean Value
Illumination Conditions	Artificial Light Source	Natural Light	Artificial Light Source	Natural Light	
BLIINDS2 [20]	0.18	0.68	0.60	0.72	0.55
BRISQUE [18]	0.38	0.66	0.78	0.69	0.63
CORNIA [25]	0.39	0.17	0.24	0.07	0.22
DIIVINE [17]	0.15	0.49	0.64	0.69	0.49
Grad_Log [21]	0.23	0.52	0.28	0.17	0.30
HOSA [26]	0.52	0.72	0.58	0.68	0.63
ILNIQE [22]	0.61	0.35	0.76	0.52	0.56
MsKLT [28]	0.27	0.01	0.38	0.50	0.29
SNPNIQE [23]	0.41	0.47	0.71	0.75	0.59
SSEQ [27]	0.10	0.60	0.49	0.09	0.32
NPQI [24]	0.24	0.53	0.58	0.70	0.51
UCIQE [37]	0.48	0.63	0.40	0.86	0.59
UIQM [36]	0.08	0.65	0.75	0.65	0.53
FDUM [38]	0.21	0.59	0.60	0.91	0.58

**Table 6:** PLCC between each method and $\Delta {E^{*}}_{\mathrm{ab}}$ under different experimental conditions.

Water Transparency	325 cm	325 cm	182 cm	182 cm	85 cm	Mean Value
Illumination Conditions	Artificial Light Source	Natural Light	Artificial Light Source	Natural Light	Artificial Light Source	
ILNIQE [22]	0.59	0.77	0.21	0.85	0.95	0.67
MsKLT [28]	0.53	0.73	0.50	0.85	0.54	0.63
UCIQE [37]	0.12	0.67	0.97	0.98	0.98	0.74
UIQM [36]	0.11	0.66	0.82	0.99	0.92	0.70
FDUM [38]	0.39	0.68	0.58	0.98	0.28	0.58

**Table 7:** Judgment results of each evaluation method on 8 pairs of images in the UIEB database.

Method	(a)	(b)	(c)	(d)	(e)	(f)	(g)	(h)	Accuracy
DIVINE [28]	F	F	F	F	T	T	T	T	50%
BRISQUE [18]	T	T	T	T	T	F	F	T	75%
BLIINDS2 [20]	F	T	T	T	F	F	F	F	37.5%
Grad_Log [21]	T	T	T	F	T	T	T	T	87.5%
ILNIQE [22]	T	T	T	T	T	T	T	T	100%
SNPNIQE [23]	F	F	F	F	T	T	T	T	50%
SSEQ [27]	F	F	T	F	T	T	T	T	62.5%
MsKLT [28]	F	F	T	F	T	T	T	T	62.5%
CORNIA [25]	F	F	F	T	T	T	T	T	62.5%
HOSA [26]	F	T	T	F	T	T	T	T	75%
UCIQE [37]	T	T	T	T	T	T	T	T	100%
UIQM [36]	T	T	T	T	T	T	T	T	100%
FDUM [38]	T	T	T	T	T	T	T	T	100%

## Data Availability

The TankImage-I dataset is publicly available on https://github.com/JOU-UIP/TankImage-I, accesed on 15 March 2022.
